# Life-threatening obsessive–compulsive disorder precipitated by the COVID-19 pandemic in an adolescent

**DOI:** 10.1192/bjb.2022.32

**Published:** 2023-10

**Authors:** Ahrane Jayakumar, Javier Sanchez-Cerezo, Afshan Khayam, Brigitte Spreeuwenberg, Matthew Hodes

**Affiliations:** 1 Central and North West London NHS Foundation Trust, UK; 2 Imperial College London, UK; 3 Enfield Child and Adolescent Mental Health Services, UK

**Keywords:** obsessive–compulsive disorder, COVID-19, in-patient treatment, children and adolescents, exposure response prevention

## Abstract

The COVID-19 pandemic starting in 2020 has had massive mental health consequences worldwide. It has caused generalised fear and anxiety about catching, spreading and suffering from the virus. This article describes a fictionalised patient's presentation of life-threatening obsessive–compulsive disorder (OCD) associated with fears of catching COVID-19. The fears resulted in refusal to eat and drink, with subsequent weight loss that required paediatric admission. The scenario portrays the association between COVID-19 and life-threatening OCD symptoms and goes on to illustrate the patient's good response to standard OCD treatments.

## Clinical scenario

You are a core psychiatry trainee in a child and adolescent mental health service (CAMHS) team, working in a paediatric liaison service at the local general hospital. You have been notified that a new patient, AB, has been admitted to the paediatric ward owing to weight loss and refusal to eat and drink, and appears in an extremely agitated state. He was clinically dehydrated with mild renal impairment and was given intravenous fluids for rehydration therapy. He has now been medically cleared by the paediatric team and had an unremarkable electrocardiogram, normal blood glucose levels and normal remaining blood test results. You have been advised to assess the patient on the ward.

When you meet the patient, you find that he is extremely agitated as he feels unsafe in the ward environment owing to his crippling fear of touching contaminated objects or surfaces. His mother provides a thorough collateral history.

AB is a 12-year-old male with a 7 month history of progressive, intrusive and distressing thoughts around COVID-19. He is preoccupied with these thoughts for 95% of the day. His mother reports that initially he was researching the virus on the internet, watching multiple news stations that focus on mortality statistics worldwide. This was followed by AB carrying out cleaning rituals. For several months, AB would spend 7–8 h washing his hands, kitchen appliances and his bedroom. His mother has also been carrying out these rituals to avoid AB becoming distressed. As a result, his mother has been struggling to attend to her other children's needs. AB had become increasingly hostile, accusing family members of being unclean and exposing him to severe acute respiratory syndrome coronavirus 2 (SARS-CoV-2). AB could not tolerate living in his family home owing to his paralysing fear of the virus. In the space of 3 weeks, he has moved between different homes of friends and relatives. During this period, he has been refusing to attend school, eat, drink or sleep, and has lost around 4 kg, which finally prompted attendance at the emergency department.

AB was fit and well prior to the national lockdown during March–June 2020, and he had no background of medical or psychiatric illnesses. Although he was known to have an anxious personality, he was also known to be very sociable and popular with a large group of friends at school. AB achieved the normal developmental milestones and did not experience problems at school throughout his childhood. There is no known family history of mental illness, but AB's father was diagnosed with terminal cancer prior to lockdown and became increasingly unwell.

While on the ward, AB demonstrates difficulty sustaining attention and poor memory, associated with a chronic state of starvation and insomnia. He remains preoccupied with overvalued ideas that his surroundings are contaminated by SARS-CoV-2, ultimately leading to fear of dying. He feels compelled to repeatedly clean his hands and surrounding surfaces to neutralise these distressing thoughts. He showed a significant degree of avoidance of using bathroom facilities and eating meals freshly served on the ward.

To assess the severity of AB's OCD symptoms, the children's Yale–Brown Obsessive-Compulsive Scale (CY-BOCS) was used.^1–4^ AB scored 36/40, which reflects his severe presentation. Table 1 illustrates a breakdown of his CY-BOCS score.

Box 1 Key questions and considerations to explore with this case

- How might OCD be brought on by the COVID-19 pandemic, and which psychological mechanisms may be involved in this case?
- When might in-patient psychiatric admission be necessary?
- How should this child's physical health complications be managed?
- What treatment options should be considered?

**Table 1:** A breakdown of CY-BOCS score based on AB's initial assessment

	Descriptive breakdown of CYBOCS score during initial assessment
Time occupied by obsessive thoughts	**Severe (3)** (3–8 h per day)
Interference due to obsessive thoughts	**Severe (3)** (causes substantial impairment in social or school performance)
Distress associated with obsessive thoughts	**Extreme (4)** (near constant and disabling distress/frustration)
Resistance against obsessions	**Extreme (4)** (completely and willingly yields to all obsessions)
Degree of control over obsessive thoughts	**No control (4)** (experienced as completely involuntary, rarely able to even momentarily divert thinking)
Time spent performing compulsive behaviours	**Extreme (4)** (more than 8 h per day performing compulsions)
Interference due to compulsive behaviours	**Severe (3)** (causes substantial impairment in social or school performance)
Distress associated with compulsive behaviour	**Severe (3)** (prominent and very disturbing increase in anxiety/frustration if compulsions interrupted)
Resistance against compulsions	**Extreme (4)** (completely and willingly yields to all compulsions)
Degree of control over compulsive behaviour	**No control (4)** (no control, drive to perform behaviour experienced as completely involuntary and overpowering, rarely able to delay activity)

## Discussion

### Overview of OCD and the COVID-19 pandemic

OCD is a psychiatric disorder characterised specifically by periods of obsessions and/or compulsions, affecting 1–3% of children and adolescents.^5^ Episodes can be relapsing and remitting, occurring throughout the entire lifespan. The clinical features include intrusive thoughts and impulses, causing distress to the individual and affecting daily functioning.^1^ Given the underlying neurobiological abnormalities found in young people with OCD, many have argued that this is a neuropsychiatric disorder, although the content of obsessions and compulsions may still be shaped by developmental and sociocultural processes.^6,7^

Common themes of obsessions include sexual, religious or violent thoughts around contamination from germs, dirt or bodily waste products. Compulsive behaviours can include hand-washing, cleaning rituals, and checking or counting.^1,8^

The outbreak of COVID-19 has led to a public health crisis worldwide. The UK had three national lockdown periods, which had a substantial impact on children and adolescents. Many families experienced bereavement, unemployment and financial hardship. Children and adolescents were also affected socially owing to school closures.^5,9,10^

The World Health Organization published guidance on hand hygiene, which was adopted and made the subject of a campaign in the UK as part of its guidance on infection prevention, along with advice on social distancing and mask use.^11–13^

A recent paper described the changes in OCD phenomenology and presentation in the context of COVID-19.^14^ Table 2 illustrates the convergence of hygiene behaviours advised during the pandemic and behaviours observed in some patients with OCD during this period.^14^

**Table 2:** Potential similarities and differences between COVID-19 government guidelines and behaviours due to OCD

COVID-19 government guidelines	OCD
Wash hands or use hand gel for 20 s	Wash hands or use hand gel until ‘internal referenced’ criterion has been satisfied, likely to be more than 20 s
Wash hands or use hand gel when returning home from public place	Wash hands or use hand gel in response to an obsession, idiosyncratic trigger or pre-occupation/obsession regarding COVID-19 (likely to be frequent and excessive)
Wash hands or use hand gel generally following steps advised by government	Wash hands or use hand gel in a rigid and ritualised way
Avoid touching things others may have touched things others may have touched – use sanitiser	Wear gloves at all times, even at home
Do not allow anyone outside family in home	Do not allow anyone in the home
Social distance	Stop going out altogether

Originally from Jassi et al 2020.^14^ The format of the table has been altered although the content remains the same.

The impact on the mental health of many children and adolescents with OCD has been well documented during the pandemic.^15,16^ A significant increase in CAMHS referrals has been noted compared with pre-pandemic periods.^15–17^ A recent systematic review investigating the association between the pandemic and exacerbation of OCD revealed a significant correlation between the two.^17–20^

## Assessment of OCD

### Formulation

For suspected cases of OCD, it is crucial to perform a thorough clinical evaluation.^1^ This includes taking a focused history to establish the duration and impairment of symptoms, assessment of comorbidities, and obtaining a collateral history from relevant parties, such as the primary caregiver (usually the parent) and school teachers.

In this case scenario, AB had a complex presentation with apparent psychological and social factors influencing the onset and perpetuation of his OCD. In these cases, it is useful to construct a thorough formulation. As an example, Table 3 summarises the predisposing, precipitating, perpetuating and protective factors related to the development of AB's OCD.

**Table 3:** Visual representation of AB's formulation

Factors	Child	Family	Environment
Predisposing	Anxious personality type	Mother has anxious personality traits	Overcrowded house Shared bedroom with younger siblings
Precipitating	Child's appraisal of COVID-19	Father becoming severely unwell, diagnosed with terminal illness during lockdown period.	COVID-19 pandemic and national lockdown School closure
Perpetuating	Poor oral intake and weight loss. Body in starvation can impair cognition. Safety-seeking behaviours including harm avoidance, compulsive hand-washing and cleaning rituals which would negatively reinforce his beliefs around contamination Over-inflated responsibility for passing on SARS-CoV-2 to his family	Family accommodation, e.g. mother following AB's cleaning rituals for several hours to avoid AB becoming distressed Mother buying three meals every day while AB was in hospital Father's illness Maternal burden	News outlet and social media broadcasting regular updates on the status of COVID-19 including mortality rates, health risks and hygiene/hand-washing campaigns Healthcare staff on the ward reinforcing his behaviour by supplying his alcohol gel, moving objects perceived as contaminated
Protective	Positive attitude to treatment Maternal boundaries Good peer relationships	Positive attitude to treatment Supportive mother Close relationship with his siblings	School has been supportive

### Distinguishing OCD from a psychotic disorder

AB's presentation may easily be confused with psychosis or delusional disorder as his ability to think rationally is temporarily impaired when challenged with a stressor such as touching the bathroom taps owing to his arousal state, and his distorted cognitions reach delusional intensity. However, after the stressful situation, his ability to think rationally returns and he is able to acknowledge that catching the virus is not a certainty. By contrast, in the case of a delusion, the fixed false belief would be unshakable despite evidence to disprove it. AB believes these thoughts to be originating inside his head.

It is important to bear in mind that if a patient with severe OCD is highly aroused, he or she may not admit to the irrationality of the beliefs, but the presence of compulsions, lack of other symptoms of psychosis and the history of development of symptoms may also point to OCD.

### Management of OCD in an in-patient setting

Admission of a child due to OCD is rare. In this case, admission was necessary in order to safely assess, monitor and manage AB's acute risk to physical health, nutritional status, weight and sleep regime. Ideally, AB would have been transferred to a child and adolescent mental health unit for intensive OCD treatment; however, there were no available mental health beds to accommodate this. Therefore, AB remained on the paediatric ward until he reached stability and was reviewed by the paediatric liaison psychiatry team four times a week. OCD treatment was commenced and monitored by the liaison team. Psychiatric admission can also be used in a planned management approach for intensive cognitive–behavioural therapy (CBT) in severe treatment-refractory OCD, especially when it may be helpful to remove a child from the family.

#### Management of physical health

Any young patient in a critical state of starvation runs a dangerous risk of severe hypoglycaemia, coma and death.^21^ There is also a high risk of refeeding syndrome when reintroducing dietary intake. Hypokalaemia and hypophosphataemia may be seen in these cases, so urgent blood tests are required to identify these electrolyte and metabolic abnormalities.^22^ Failure to recognise these may result in emergency complications such as arrhythmias and seizures.^21,23^

Immediate life-threatening risks must be managed accordingly, and frequent monitoring of vital signs is also imperative in overall management. A dietician should be contacted to help prescribe a safe meal plan to minimise the risk of refeeding syndrome in a starved patient.

#### Treatment of psychiatric symptoms

Once physical health has been stabilised, it is easier to focus on acute psychiatric symptoms on the ward. The National Institute for Health and Care Excellence (NICE) suggests that it would be helpful to use a standardised rating scale for children, adolescents and adults to assess their symptoms and level of functionality throughout the treatment period.^24^ CY-BOCS is a robust and validated rating tool used to assess the severity of OCD symptoms in children and adolescents.^1–4^ In this case, CY-BOCS was used throughout AB's admission to guide management and evaluate his response to combined pharmacological and psychological treatment interventions.

NICE recommends combination treatment with CBT and selective serotonin reuptake inhibitor (SSRI) medication for severe OCD cases in anyone above the age of 8 years old.^24,25^. In the UK, for children and adolescents, sertraline, fluvoxamine and clomipramine are licenced for OCD treatment.^25^ NICE recommends SSRIs as first-line treatment and clomipramine as second-line treatment, owing to potential adverse effects and tolerability of tricyclic antidepressants. Additional SSRIs are licensed for OCD treatment in adults, including fluoxetine and paroxetine.^24^ In children, medications are started at a low dose appropriate to age and size and titrated up slowly, considering the possibility of poor tolerability and adverse effects. It is also worth noting that there is still relatively limited understanding of the long-term effects of psychotropic medication on the developing brain. In comparison to treatment for depression, the time taken to respond to treatment for OCD in adults and children is generally longer and can be up to 12 weeks.^24^

For young people with severe or resistant symptoms of OCD, treatment can be augmented with risperidone (an atypical antipsychotic).^24,25^ In this case, AB was started on low-dose sertraline, 25 mg once daily, with the dose slowly titrated to 150 mg once daily, based on weekly mental state reviews and symptom severity. Owing to AB's highly aroused and agitated state, risperidone was commenced at 0.5 mg once daily and titrated to 1.5 mg once daily based on level of agitation and response to treatment. The use of risperidone, a dopamine blocker, helped to alleviate AB's agitation over time. This subsequently made it easier for the patient to engage with our team and with exposure and response prevention (ERP) and to adhere to sertraline treatment. It was also expected that the risperidone would increase eating and drinking, known ‘side-effects’ of risperidone but desired effects in this case. The use of therapeutic-dose sertraline eventually led to symptom remission by 4 weeks on the ward. AB's global level of functioning significantly improved.

CBT includes both behavioural therapy in the form of ERP and cognitive therapy. This combination of elements of intervention unravels core beliefs behind distressing thoughts, connecting such thoughts to behaviours and essentially challenging these unhelpful thoughts to enable the patient to develop an alternative understanding of the problem. In this case, AB had obsessive thoughts of surrounding surfaces and objects being contaminated with SARS-CoV-2, driven by fear of being contaminated and dying from the infection and an intolerance of uncertainty to catching SARS-CoV-2. This brought on excessive feelings of anxiety leading to compulsive washing, which lasted for a prolonged duration until AB felt right, as well as harmful avoidant behaviours such as not eating food prepared by others. Although these behaviours relieved his anxiety, this was a temporary effect as the obsessions around contamination would return, creating vicious cycles of thoughts, feelings and behaviours.

ERP involves working with the patient so that they can produce a list of situations they find anxiety-provoking. Early in therapy, the therapist teaches skills to manage anxiety, such as breathing exercises and relaxation techniques. The patient and therapist will work through each situation together, exposing the patient to a stressful stimulus and challenging negative cognitions until the patient feels completely comfortable, before moving on to the next situation. Eventually, the patient will learn not to associate certain stimuli with irrational beliefs, reducing anxiety levels.^27^

Both the specialist registrar and the therapist from the liaison team introduced and delivered the ERP intervention on the ward, three times a week. They also educated nursing staff on the ward so they could assist with ERP three times daily. AB was encouraged to touch perceived contaminated objects on the ward, without engaging in safety-seeking behaviours such as asking for assistance, and access to soap, alcohol gel and cleaning wipes was removed. Examples of challenges included touching door handles, eating meals provided on the ward and allowing nursing staff to apply a blood pressure cuff on his arm. Initially, AB became preoccupied with the boundaries implemented on the ward and the transfer of germs onto his body, which led to further avoidance, agitation and multiple attempts to negotiate with nursing staff to regain access to soap and alcohol gel. Over repeated exposures, regular assistance from nursing staff in ERP and observing staff modelling healthy behaviours on the ward, AB slowly began to feel less anxious about catching COVID-19 and more comfortable on the ward.

Family involvement and psychoeducation have important roles in CBT treatment, as they address accommodation of safety-seeking behaviours such as taking part in rituals to reduce the affected patient's anxiety levels. In this case, AB's mother had been repeatedly supplying large volumes of alcohol gel and assisting AB to use the toilet facilities so that AB would not come into contact with various surfaces. She also provided emotional support without discussing content of his fears. This was perpetuating AB's illness, reinforcing his bizarre behaviours.

Standard treatment for OCD as recommended by NICE, based on severity of symptoms and impact on functionality, remains the foundation to follow, including when dealing with new cases during the COVID-19 pandemic. The COVID-19 pandemic is an example of a major life stressor. The pandemic alone is not a causative factor of OCD; however, it is associated with an increase in new presentations of children and adults with OCD. Additional research is required to further understand the relationship between the COVID-19 pandemic and the presentation of OCD symptoms, and to establish optimal treatment pathways after the pandemic.^28^ In this case, AB's symptoms were uniquely related to COVID-19 and potentially precipitated by the effects of the pandemic. Nevertheless, AB's symptoms improved significantly and responded well to the standard treatment for OCD.

### Working closely with patients, families and multidisciplinary teams

In the treatment of OCD in children and adolescents, it is important that healthcare providers are collaborative and engage with the family. It is not uncommon that parents are deeply engaged in behaviours that accommodate their child's distress. By providing immediate relief, they may inadvertently reinforce the cycle of obsessions and compulsions.^29^ In this case, AB's mother was performing actions that permitted him to avoid the feared situations. For example, she was bringing to the ward only certain types of food that AB thought that were not contagious. It was of vital importance to involve his mother in the treatment. We used a collaborative approach with AB and his mother, letting them be involved in the decision-making and the treatment plan. AB's mother helped to slowly increase the levels of exposure in his environment and therefore aided the process of recovery.

Psychoeducation had a vital part to play in this case. Relevant information about OCD was often provided, ensuring that information was correctly understood by the patient and his mother. When providing information to a young person, this should be done according to their level of development. For this case, we used an ‘externalisation’ approach model. First, we separated the young person from the disorder; second, we avoided blaming him for what was happening; and, third, we always foregrounded optimism in the recovery.

Although the diagnosis of OCD in young people is similar to that in adults, an important distinction is insight. Children are less likely to have insight into the irrationality of their obsessions and compulsions.^30^ Initially, AB had poor insight and thought that his obsessive–compulsive beliefs were probably true; thus, his obsessions were ego-syntonic rather than ego-dystonic when he was exposed to anxiety-provoking situations. This later reversed when the patient calmed down and moved away from specific triggers.

Liaising with the paediatricians and other professionals including dieticians was another fundamental aspect in the management of this case. It is vital that a multidisciplinary approach is used in the treatment of paediatric patients with complex physical and mental health problems.

### Prognosis

Outcomes are often more favourable and treatment responses are more robust and durable in paediatric patients with OCD compared with adults.^29^ However, longer duration of illness, resistance to treatment, poor engagement and adherence, and refusal of treatment predict poorer outcomes, which include development of comorbidities such as depression, impaired functionality and poorer quality of life. Refusing or prematurely ending treatment may be due to extreme fear of facing the consequences of performing rituals or compulsions.^24^

### Patient's perspective

An anonymous patient with similar lived experience has shared a few comments about their experience:

‘I didn't know I had OCD. Last year during the pandemic, I genuinely felt scared and worried about that I would die from Corona virus. I thought all that cleaning and hand-washing I did was normal because the government said so on TV! I was forced to go into hospital. I felt unsafe and was sure I would catch Corona. I hated being challenged on the wards, being asked to touch things. I hated the doctors and. therapist in the beginning, but they continued to be nice and patient with me. Helped me to relax and make me feel safe. I guess over time I realised I wasn't going to die if I ate the hospital food or had a shower on the ward. Looking back, I can see I needed the treatment and that my behaviour before coming into hospital was not normal.’

## Conclusion

The COVID-19 pandemic was a unique and stressful period that affected children and adolescents in multiple ways, from socialising to coping with the stress associated with lockdown policies. Recent research has shown an increase in exacerbations of OCD in children and adolescents during the pandemic, with individuals displaying symptoms of contamination obsessions, cleaning or hand-washing compulsions. These symptoms appeared to have been aggravated by public health campaigns and media drives to promote cleanliness and infection control and/or prevention. There is currently insufficient available data to quantify the effects of the pandemic on these behaviours in the UK.

New cases of OCD in the child and adolescent population in relation to the COVID-19 pandemic can be managed following recommended treatment as per NICE guidance.^24^ This includes combination therapy of SSRI medication and ERP.

## Further information on OCD

There are various resources including websites and books for teenagers and parents to access, and to help raise awareness on OCD in general (https://ocdaction.org.uk/).^31^ Furthermore, there is an award-winning educational film called *UNSTUCK. A Kid's Movie* that also portrays insight behind the illness (https://www.ocdkidsmovie.com).

## Consent statement

No consent was required as a fictionalised case was used, but this case was derived from treating a young adolescent with similar clinical characteristics and response.

## About the authors

**Ahrane Jayakumar** is a core trainee in psychiatry at Westminster CAMHS in Central and North West London NHS Foundation Trust, UK. **Javier Sanchez-Cerezo** is a clinical fellow at Westminster CAMHS in Central and North West London NHS Foundation Trust, and in the Division of Psychiatry, Imperial College London, UK. **Afshan Khayam** is a consultant in child and adolescent psychiatry at Enfield CAMHS. **Brigitte Spreeuwenberg** is a family and systemic psychotherapist in the paediatric liaison service at Westminster CAMHS in Central and North West London NHS Foundation Trust, UK. **Matthew Hodes** is a consultant in child and adolescent psychiatry at Westminster CAMHS in Central and North West London NHS Foundation Trust, London, and honorary clinical senior lecturer in child and adolescent Psychiatry, Division of Psychiatry, Imperial College London, UK
